# School Identification Alleviates Depressive Mood Among Chinese College Students: The Chain Mediating Role of Social Exclusion and Sense of Control

**DOI:** 10.3390/bs16081445

**Published:** 2026-08-21

**Authors:** Yanqiong Zhong, Lei Yan, Jialing Liu, Yanhong Zhang, Linchuan Yang

**Affiliations:** 1 College of Education and Sports Sciences, Yangtze University, Jingzhou 434023, China; 2024710087@yangtze.edu.cn (Y.Z.); 2023710073@yangtze.edu.cn (J.L.); 2 Social Psychology Research Center, Yangtze University, Jingzhou 434023, China; zhangyh@yangtzeu.edu.cn; 3 College of Education, Three Gorges University, Yichang 443002, China; yanglinchuan@ctgu.edu.cn

**Keywords:** school identification, depressive mood, social exclusion, sense of control

## Abstract

Depression represents a growing public health concern globally, with the mental health of college students requiring targeted attention. Drawing on the social cure theory, self-determination theory, and the agency-communion model, the current study explored the mechanisms linking school identification to depressive mood among Chinese college students. Specifically, this cross-sectional study aimed to investigate the association between school identification and depressive mood, and whether social exclusion and sense of control mediate this relationship. A sample of 981 college students (464 males and 517 females) completed self-reported measures of school identification, depressive mood, social exclusion, and sense of control. Results showed that (1) School identification was negatively associated with college students’ depressive mood. (2) School identification affected depressive mood through three significant indirect pathways: the single mediating path via social exclusion, the single mediating path via sense of control, and the chain mediating path from social exclusion to sense of control. These findings reveal that the mechanism by which school identification alleviates depressive mood lies in first providing communion resources (reducing social exclusion) and then enhancing agency resources (increasing sense of control), thereby providing empirical support for the agency-communion model and offering practical implications for educational interventions targeting college students’ depressive mood.

## 1. Introduction

Depression is a common emotional problem among young people, with college students being particularly vulnerable (X. Liu et al., 2023). Epidemiological data indicate that the prevalence of depression risk in the 18–24 age group reaches 24.1% (Fu et al., 2023). A recent meta-analysis of 32 studies involving 93,679 Chinese university students reported a pooled depression prevalence of 34.70%, with rates increasing during and after the COVID-19 pandemic (Lin et al., 2025). In particular, first and second-year college students exhibit higher levels of depressive symptoms compared to their senior counterparts, as they are navigating the critical transition from adolescence to adulthood while adapting to a new academic and social environment (Fang et al., 2025). College students generally face academic, interpersonal, and personal growth pressures, which may induce depressive mood. Depressive mood is a mild form of depression typically characterized by anhedonia and negative emotions (Gong et al., 2010). Without timely intervention, sufferers are highly likely to develop more severe depressive syndromes and disorders. Given its high prevalence, depression among college students can significantly impair key life domains, including physical health, psychological adjustment, academic performance, and social interactions. Therefore, identifying potential protective factors against college students’ depressive mood and their internal mechanisms has great practical significance.

In this context, numerous researchers have examined the individual physiological and psychological factors influencing depressive mood among college students. Psychologically, early-year undergraduate students are typically in late adolescence. Dependence on family and insufficient self-reliance can impair their adjustment to campus life, raising the risk of developing psychological concerns such as obsessive symptoms, depression, and anxiety. In terms of psychological factors, self-identity, cognitive style, attributional style, and personality traits can affect mental health. For instance, pessimistic attributional styles and ruminative thinking are likely to cause psychological problems in college students (Lo et al., 2010; Luo et al., 2024). Overall, previous studies on depressive mood among college students have focused more on individual factors and less on group perspectives.

Previous studies have found that social identity formed against the backdrop of different group identities is a significant predictive factor in alleviating individuals’ depressive mood (Seymour-Smith et al., 2017), and school identification is one of the most vital social identities for college students. First, from the perspective of social identity theory, group membership and identity play a key role in personal psychological development (Tajfel & Turner, 1979). A review of social identity and group connections demonstrated a positive relationship between group identity and psychological well-being (Haslam et al., 2022). Extending this framework to educational settings, school identification may have a similar positive impact on individuals’ mental health. Conceptually, school identification refers to an individual’s sense of belonging to their school, coupled with positive valuation of the school and its outcomes (Voelkl, 1997). According to Erikson’s definition of the eight psychosocial development stages, undergraduate students in late adolescence are characterized by core tasks of self-definition, clarifying group roles and status, and consolidating personal identity. In this context, school identification provides students key psychological resources, such as belonging and perceived value, that support the formation of adaptive self-concepts (Voelkl, 2012). In addition, the school environment represents a foundational developmental context and significantly shapes college students’ psychological adjustment (Tong et al., 2019). Research has shown that diminished school climate and weakened school identification are associated with reduced well-being, poorer mental health, and lower academic engagement, likely due to the erosion of meaningful group connections (Chen et al., 2024). In conclusion, these findings underscore the value of examining how school identification relates to depressive mood among college students from a group-level perspective.

Although a negative association between school identification and depressive mood has been demonstrated, the underlying mechanisms through which this association operates remain underexplored. Reviews of social identity and depression have pointed out that social connection is central to understanding both the onset and recovery from clinical depression (Cruwys et al., 2014). The sense of belonging, as a positive effect of social connection, has received widespread attention from researchers (Jiang et al., 2025). Nevertheless, prior studies have often failed to consider the range of risk factors related to social connection, such as social exclusion. College students may experience life events such as poor social adaptation, academic pressure, emotional distress, personal, physical, and mental defects, and dissatisfaction with one’ s major, all of which trigger their depressive mood. Compared to the abstract concept of sense of belonging, social exclusion involves specific behaviors and events and is easier to observe. Thus, the present study sought to examine whether social exclusion mediates the association between school identification and depressive mood among college students. Furthermore, good self-control is a key factor in promoting adaptation for freshmen (J. B. Li et al., 2023). The agency-communion model explains how social identity influences depression (Yan et al., 2023), which categorizes psychological resources that meet different needs into two major types: communal and agentic resources. Communal resources refer to psychological resources for maintaining connections with others, such as perceived social support and sense of belonging, while agency resources facilitate the functioning of individuals as independent entities, with a sense of control. College students with poor interpersonal relationships have problems seeking help from the outside world when facing difficulties. Their sense of control is weakened, which may induce a depressive mood. School identification may alleviate social exclusion by providing communal resources and agentic resources (sense of control) to mitigate college students’ depressive mood. Therefore, this study advances our understanding of the mechanisms linking school identification to depressive mood, by examining the mediating roles of social exclusion and sense of control.

### 1.1. The Relationship Between School Identification and Depressive Mood

School identification can provide a series of psychological resources to alleviate college students’ depressive mood. First, school identification as a type of social identity may be an important factor in improving college students’ depressive mood. Social cure theory posits that group-based social identity shapes mental health by modifying individuals’ cognitive appraisals and behavioral patterns (Jetten et al., 2017). Group membership can provide individuals with psychological resources such as connectedness, meaning, efficacy, and social support. For college students, being a college student is their primary and most important group identity, which is related to how they view and handle themselves and others (Bowman & Felix, 2017). Therefore, we infer that school identification can alleviate depressive mood among college students.

Second, school identification refers to the sense of acceptance and respect felt in the school environment, belonging, and value for the school. The social identity model of depression posits that depressive states arise when individuals lack key psychological resources derived from group membership (Cruwys et al., 2014). Drawing on this theory, previous researchers have linked school identification with depression and found that school identification influences depression in college students. For instance, a three-wave longitudinal study showed that school identification can significantly predict students’ well-being dimensions such as positive affect and depression (Klik et al., 2023). Studies have also demonstrated that school identification yields stronger mental health benefits for students than identification with family or peer groups (Miller et al., 2018). Therefore, school identification can offer a sense of belonging and strengthen connections with the group, thereby alleviating college students’ depressive mood. In conclusion, the current study advances the following hypothesis:

**Hypothesis** **1.** *School identification will be negatively related to depressive mood among college students.*

However, existing literature has rarely examined the relationship between school identification and depressive mood in college students, and the underlying mechanisms remain underexplored. To address this gap, the current study sought to systematically examine how school identification relates to depressive mood from a social identity perspective.

### 1.2. Social Exclusion as a Mediator

Within the literature on depressive symptoms among college students, sense of belonging has attracted the attention of scholars, while far less focus has been directed toward the role of social exclusion in shaping these outcomes. First, existing studies have repeatedly demonstrated that a lack of sense of belonging is positively correlated with level of depression (Cruwys et al., 2015; X. Wang et al., 2014). Therefore, as a key component of school identification, belonging helps explain how these group ties shape students’ depressive mood. However, when early-year undergraduate students have not yet formed school identification, they lack a sense of inclusion within the school community and may thus be more vulnerable in interpersonal contexts. College students are vulnerable to offline and online social exclusion, which can lead to video game addiction through depression (K. P. Li et al., 2024). Therefore, this study holds that incorporating social exclusion into research on college students’ depressive mood is of great significance.

Social exclusion describes the experience and process in which an individual’s need for belonging and social connection is frustrated by rejection or exclusion from a social group (Du & Xia, 2008). Social exclusion among college students can be summarized as a marginalization phenomenon in which college students cannot maintain or establish normal interpersonal relationships in campus life and groups, are not recognized, and lose the right to participate. The temporal need-threat model focuses on the psychological and behavioral responses of individuals who experience exclusion (Williams, 2009). When individuals are excluded, their basic psychological needs (sense of belonging, control, presence, and self-esteem) are threatened, leading to emotional reactions such as anxiety and depression. Students who perceive themselves as socially excluded may be at a disadvantage academically and socially, experiencing strong feelings of loneliness and negative affect (C. Zhang et al., 2023).

In addition, a high level of school identification among college students may be associated with lower levels of social exclusion. As mentioned earlier, in social cure theory (Jetten et al., 2017), school identification can bring individuals a sense of belonging and social support and increase communal resources, thereby alleviating the spread of depressive mood. Research has shown that social support can be an effective and positive resource for alleviating the negative emotions from being rejected (Morese et al., 2019). Moreover, self-categorization theory suggests that people automatically categorize things and distinguish others into in-groups and out-groups. Individuals also include themselves in these categories and attribute the characteristics of the in-group to themselves (Turner et al., 1987). Students reporting stronger school identification will uphold the school’s values and educational philosophy, comply with school rules and regulations, actively adapt to the school environment, and demonstrate greater learning engagement and proactive, serious learning behaviors in their studies. Thus, individuals who identify more closely with their school tend to gain recognition from teachers, classmates, and the group, reducing the likelihood of being excluded by others.

Furthermore, scholars have noted that developmental psychology research lacks exploration of social exclusion from the perspective of group identity (Abrams & Killen, 2014). Students can gain sense of belonging and security from meaningful connections with the group, thereby alleviating their depressive emotions. Meanwhile, existing empirical studies have considered workplace exclusion as a mediating variable to examine its impact on self-harm behaviors and sense of happiness (C. Z. Liu et al., 2020). Taken together, the evidence reviewed leads to the following hypothesis:

**Hypothesis** **2.** *School identification may reduce college students’ depressive mood via the mediating role of diminished social exclusion.*

In addition to environmental external factors, the influence of individual internal factors on depressive mood has also attracted the attention of researchers. Studies have confirmed that one’s sense of control acts as a robust determinant of overall health, influencing both physical functioning and psychological well-being (C. Cheng et al., 2013). Thus, this study analyzed sense of control as a mediator to clarify the mechanism of school identification and depressive mood.

### 1.3. Sense of Control as a Mediator

Sense of control refers to individuals’ subjective perception of whether and to what extent we can master events (Yao & Ren, 2023). College students often face adaptation problems, such as interpersonal distress, academic pressure, personal emotional adjustment, and goal setting. They cannot adapt quickly to environmental changes, leading to confusion and helplessness. Self-determination theory states that individuals have three fundamental psychological needs: autonomy, competence, and relatedness (Deci & Ryan, 2013). The essence of sense of control lies in the satisfaction of the need for autonomy. Specifically, when individuals perceive that their behavior is driven by their own will, their sense of control is strengthened. Conversely, this may lead to depression (Campbell et al., 2018).

School identification can provide college students with an agentic resource, namely, a sense of control, which promotes mental health. As outlined by self-categorization theory, self-stereotyping following the salience of group identity prompts individuals’ cognitions to align with the group, prompting them to examine themselves and envision the future from the collective perspective (Turner et al., 1987). That means, individuals can not only get a sense of belonging through group identification but also share the meaning and goals of the group. For example, individuals with a higher level of community identity believe they can utilize community resources, which enhances their resilience against uncertain events in life (Y. Wang et al., 2021). Consistent with this perspective, school identification can provide college students with emotional support and psychological security. When students identify with a school as a group and develop a sense of school identification, they internalize the school’s success and its positive aspects as part of themselves. This, in turn, enhances their perception of their own abilities and improves their self-regulatory capacity.

Furthermore, existing work further highlights sense of control as a key mechanism explaining how social identity shapes mental health outcomes (Greenaway et al., 2015; Yan et al., 2023). As established in previous research, it functions as a robust mediator of well-being. For college students, stronger school identification is associated with greater access to institutional and interpersonal resources, which can foster a stronger sense of control and reduce depressive mood. Based on this inference, we propose Hypothesis 3:

**Hypothesis** **3.** *The association between school identification and reduced depressive mood in college students is mediated by a higher sense of control.*

### 1.4. Social Exclusion and Sense of Control as Serial Mediators

The link between social exclusion and sense of control is well-established. First, as the temporal need-threat model posits, exclusion undermines psychological needs, particularly control, which in turn elevates depression risk (Williams, 2009). When sense of control is diminished by exclusion, individuals are more prone to experiencing negative affect and engaging in harmful behaviors (S. Li et al., 2021; X. L. Yang et al., 2025). Second, social exclusion can increase individuals’ experience of negative emotions through the mediation of sense of control. Third, the agency-communion model of how social identity influences depression suggests that identification first provides communal resources, then offers agentic resources, and ultimately influences individuals’ depressive emotions (Yan et al., 2023). In the current study, school identification helps college students integrate into the collective, and strengthens group connections, thereby reducing the level of social exclusion and establishing a psychological support system. This enables them to acquire agency resources through interactions with others, enhances their sense of control, and ultimately alleviates depressive mood. In summary, this research suggests that students who identify more strongly with their school experience less social exclusion and possess a greater sense of control, both of which serve as protective factors against depressive mood. We therefore propose:

**Hypothesis** **4.** *The negative association between school identification and depressive mood in college students is explained by the sequential mediating roles of reduced social exclusion and enhanced sense of control.*

### 1.5. Overview of the Present Study

**Figure 1:**
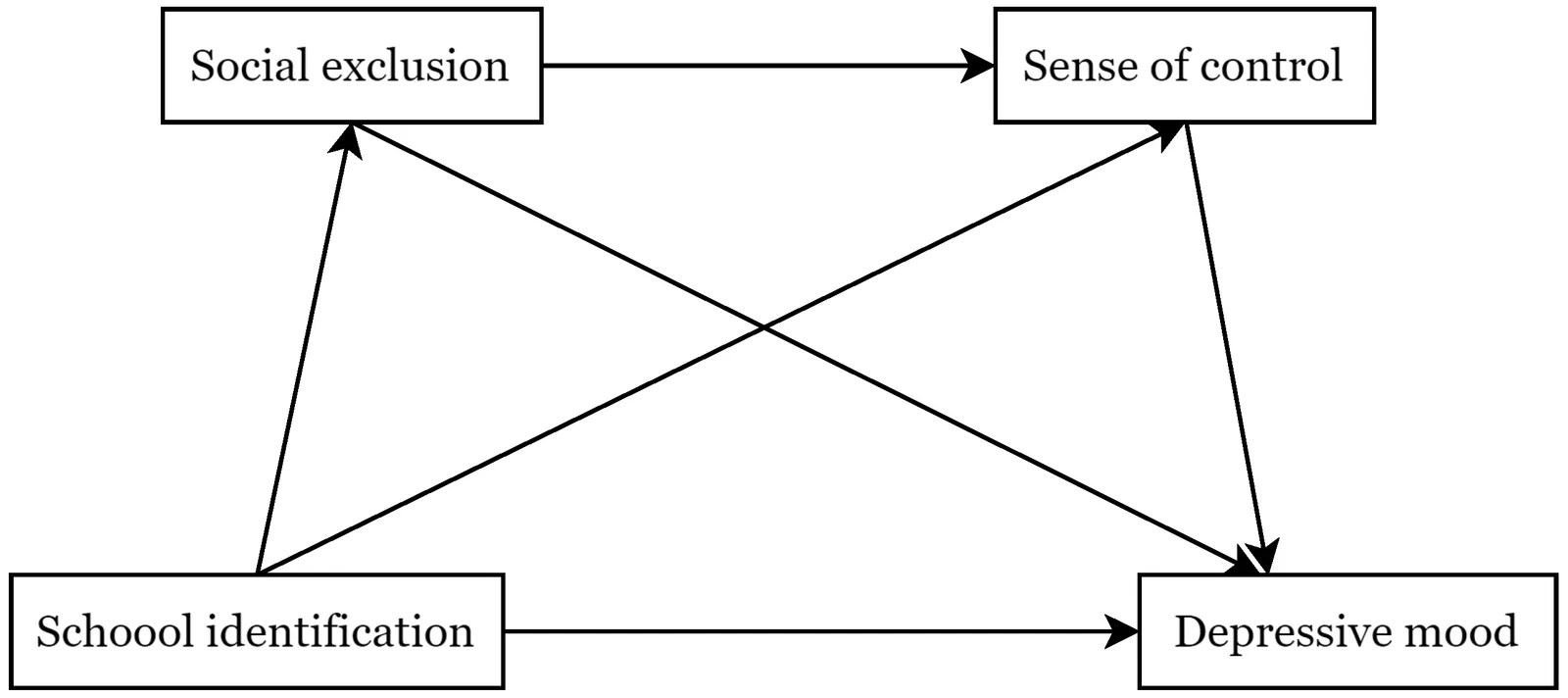
The proposed serial mediation model. →: the arrow signifies a predictive function.

The present study was designed to comprehensively analyze the direct and indirect pathways via which school identification, social exclusion, and sense of control shape depressive mood in college students. Specifically, it sought to examine the potential sequential mediating effects of social exclusion and sense of control in this relationship. The full conceptual framework is illustrated in Figure 1. Beyond clarifying the direct association between school identification and depressive mood, this research aims to uncover the underlying psychological mechanisms, thereby informing targeted prevention and intervention strategies for college students’ mental health.

## 2. Materials and Methods

### 2.1. Participants

Participants were undergraduate students recruited from a comprehensive university located in Jingzhou, a major city in central China. A total of 981 students participated in this study, consisting of 464 males (47.3%) and 517 females (52.7%). The sample was predominantly composed of lower-year students: 752 (76.7%) were freshmen, and 162 (16.5%) were sophomores. The remaining 67 participants (6.8%) were juniors and seniors. Lower-year college students are in a critical transitional period from high school to university, during which they are establishing new social relationships, adapting to a new academic environment, and developing their identification with the school. Therefore, this population provides a particularly appropriate context for examining how school identification influences depressive mood. It should be noted, however, that the present sample was predominantly composed of lower-year students, with upper-year students being substantially underrepresented.

### 2.2. Procedure

Before starting data collection, we secured ethical approval from our institution’s research ethics committee. The data gathering process was carried out between February and May 2024. A total of 1136 questionnaires were administered using two approaches: an online format (via Questionnaire stars (Questionnaire Star, Changsha, China) and social networking platforms) and an offline format (through in-person campus recruitment). Prior to completing the questionnaire, participants were informed by the researchers that all data would be used exclusively for academic research and would be handled with strict confidentiality and anonymity. Participation was completely voluntary. We encouraged participants to respond to the questionnaire honestly and independently. All participants completed the survey within 20 min. After that, researchers collected all questionnaires and expressed a small token of appreciation to the participants.

Secondly, we removed invalid questionnaires according to completion time and response quality (e.g., incomplete response, selecting the same option for every question, or careless answering). Eventually, we screened 981 valid questionnaires from 1136 questionnaires, and the valid recovery rate was 86.36%.

### 2.3. Measurements

#### 2.3.1. School Identification

School identification was measured using the School Identification Scale revised by Ding (2012). This scale consists of 20 items, covering four dimensions: group cognition (4 items, e.g., “I believe the image of my school reflects my own image to some extent”), emotional connection (5 items, e.g., “I feel proud to be a student of my school”), positive evaluation (5 items, e.g., “I consider my school to be an excellent university”), and autonomous behavior (6 items, e.g., “I strive to uphold the image of my school when I am outside”). All items were rated on a 7-point Likert scale, ranging from 1 (completely disagree) to 7 (completely agree). In this study, the Cronbach’s α coefficients for the four dimensions (group cognition, emotional connection, positive evaluation, and autonomous behavior) were 0.733, 0.879, 0.891, and 0.873, respectively, and the Cronbach’s α coefficient for the overall scale was 0.922, indicating excellent internal consistency in our sample.

#### 2.3.2. Social Exclusion

Social exclusion was measured using the Chinese version of the Ostracism Experience Scale for Adolescents (OES-A), developed by D.-h. Zhang et al. (2018). This scale consists of 11 items, comprising two subscales: rejection and ignorance. The rejection subscale contains six items (e.g., “Others often invite me to have meals with them”), all of which are phrased in a positive manner (e.g., being invited or accepted), and thus need to be reverse-scored. The ignorance subscale contains five items (e.g., “Others always ignore me”). All items are rated on a 5-point Likert scale. After reverse-scoring, all items were aligned in the same direction. In this study, the average score of the 11 items was computed as the overall social exclusion score, with higher scores indicating higher levels of perceived social exclusion. In this study, the Cronbach’s α coefficients for the rejection and ignorance subscales were 0.791 and 0.865, respectively, and the overall scale demonstrated satisfactory internal consistency (Cronbach’s α = 0.768). The mean scores for the rejection and ignorance subscales were 3.00 (*SD* = 0.72) and 2.17 (*SD* = 0.82), respectively.

#### 2.3.3. Sense of Control

Sense of control was measured using the Chinese version of the Sense of Control Scale revised by J. Li (2012). This scale consists of 12 items, comprising two subscales: personal mastery and perceived constraints. The personal mastery subscale contains four items (e.g., “My future mainly depends on myself”), and the perceived constraints subscale contains eight items (e.g., “I often feel helpless when dealing with some problems in my life”). All items were rated on a 7-point Likert scale, ranging from 1 (strongly disagree) to 7 (strongly agree). For scoring, all eight items of the perceived constraints subscale were reverse-scored so that both subscales were aligned in the same direction, with higher scores indicating a stronger sense of control. Subsequently, the total scores of the personal mastery subscale and the reverse-scored perceived constraints subscale were calculated separately, and each was standardized within our sample. The overall sense of control score was then computed by summing the two standardized scores, with higher scores reflecting a greater overall sense of control. The overall sense of control score was computed as the sum of the two standardized scores: Sense of Control = *Z* (personal mastery total score) + *Z* (reverse-scored perceived constraints total score). In this study, the Cronbach’s α coefficients for the personal mastery subscale, the perceived constraints subscale, and the overall scale were 0.748, 0.847, and 0.785, respectively.

#### 2.3.4. Depressive Mood

Depressive mood was measured using the depression subscale of the Chinese version of the 21-item Depression Anxiety Stress Scale (DASS-21), which was validated by Gong et al. (2010). This subscale consists of seven items that assess depressive experiences in the past week, including anhedonia, lack of motivation, low mood, self-deprecation, and feelings of meaninglessness. All items were rated on a 4-point Likert scale, ranging from 1 (inconsistent) to 4 (very consistent). In this study, the average score of the seven items was computed as the depressive mood score, with higher scores indicating higher levels of depressive mood. The Cronbach’s α coefficient for the depression subscale in this study was 0.871, indicating good internal consistency.

### 2.4. Data Analysis

A sequential mediation model was tested in this research, specifying school identification as the predictor variable, depressive mood as the outcome variable, and social exclusion and sense of control as the sequential mediators. All statistical analyses were conducted using IBM SPSS Statistics Version 26.0 (Version 26.0, IBM Corp., Armonk, NY, USA). Given that all data in this study were collected via self-report questionnaires, we conducted additional analyses to assess the potential threat of common method bias. Specifically, we first constructed a baseline confirmatory factor analysis model (M_1_) comprising the four theoretical constructs. We then built an alternative model (M_2_) by adding a common method factor. Comparing the two models, the changes in fit indices were as follows: Δ*χ*^2^*/df* = 0.028, ΔGFI = 0.002, ΔIFI = 0.002, ΔNFI = 0.003, ΔSRMR = 0.002, and ΔRMSEA = 0.000. All changes fell below the threshold of 0.03, indicating that the inclusion of the common method factor did not significantly improve model fit. Thus, common method bias is unlikely to be a serious concern in this study.

Following the assessment of common method bias, the analyses proceeded in several stages. First, descriptive statistics and Pearson correlation coefficients were computed to examine the means, standard deviations, and intercorrelations among all study variables. Sequential mediation effects were then examined using Hayes’ (2017) PROCESS macro for SPSS (Version 3.5, Model 6), which employs bootstrapping to generate robust confidence intervals for indirect effects. A total of 5000 bootstrap samples were utilized to construct 95% bias-corrected confidence intervals for all indirect effects. This widely used macro is recommended for mediation analyses due to its enhanced statistical power for detecting indirect effects.

## 3. Results

### 3.1. Preliminary Analyses

Descriptive statistics (means and standard deviations) and correlations for all study variables are presented in Table 1. In line with the study hypotheses, school identification was significantly negatively associated with both social exclusion (*r* = −0.389, *p* < 0.01) and depressive mood (*r* = −0.275, *p* < 0.01), and significantly positively associated with sense of control (*r* = 0.300, *p* < 0.01). Social exclusion was positively related to depressive mood (*r* = 0.411, *p* < 0.01) and negatively related to sense of control (*r* = −0.455, *p* < 0.01). In addition, sense of control showed a significant negative correlation with depressive mood (*r* = −0.425, *p* < 0.01). Finally, gender was positively correlated with sense of control (*r* = 0.130, *p* < 0.01) and negatively correlated with social exclusion (*r* = −0.094, *p* < 0.01) and depressive mood (*r* = −0.114, *p* < 0.01). Therefore, gender was included as a covariate in the subsequent chained mediation model, and gender was coded as 1 = male, 2 = female. Given the uneven distribution of grade levels in the sample (see Section 2.1), grade was not included as a covariate in the main analyses.

### 3.2. Testing for the Proposed Chain Meditation Model

**Figure 2:**
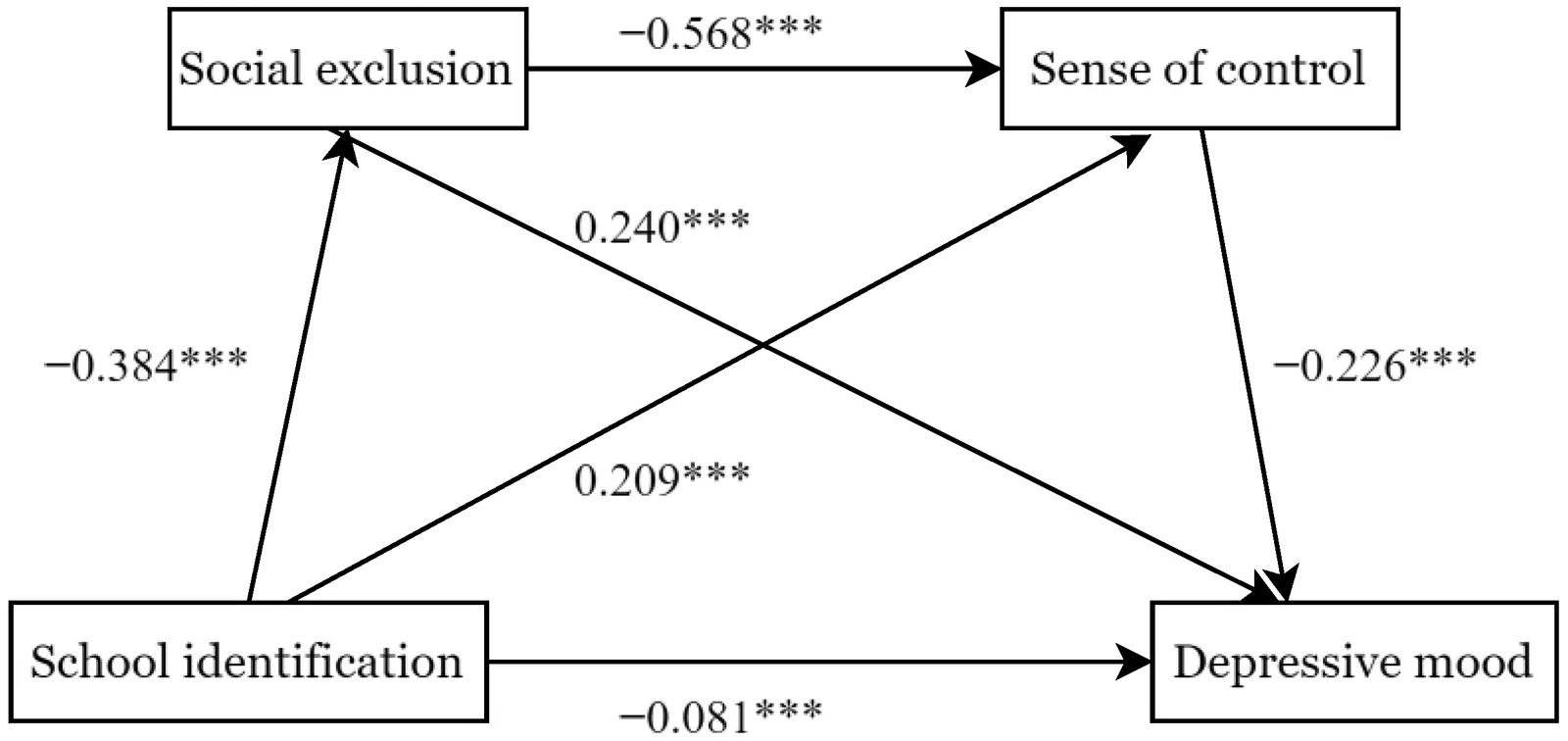
The chain mediating roles of social exclusion and sense of control in the association between school identification and depressive mood. Solid lines indicate significant paths. Values shown are standardized regression coefficients. *** p < 0.001.

Hayes’s PROCESS macro for SPSS was used to test the hypothesized chain mediation model. The primary path coefficients are illustrated in Figure 2. As shown, school identification was a significant but weak negative predictor of depressive mood (*β* = −0.081, *p* < 0.001), whereas sense of control (*β* = −0.226, *p* < 0.001) and social exclusion (*β* = 0.240, *p* < 0.001) showed small to moderate effects. Additionally, school identification was significantly and negatively associated with social exclusion (*β* = −0.384, *p* < 0.001), and positively with sense of control (*β* = 0.209, *p* < 0.001). Social exclusion, in turn, was significantly and negatively related to sense of control (*β* = −0.568, *p* < 0.001). Gender exhibited a non-significant direct predictive effect on depressive mood (*β* = −0.044, *p* = 0.106). The full mediation model accounted for 28.3% of the variance in depressive mood (*R*^2^ = 0.283).

The indirect effects of the model are presented in Table 2. The total indirect effect was significant (*b* = −0.189, 95% CI = [−0.231, −0.150]). Specifically, the indirect pathway from school identification to depressive mood via social exclusion was significant (*b* = −0.082, 95% CI = [−0.125, −0.063]). At the same time, the indirect pathway via sense of control alone was also significant (*b* = −0.047, 95% CI = [−0.074, −0.023]). Finally, the sequential indirect pathway through both social exclusion and sense of control was significant (*b* = −0.049, 95% CI = [−0.065, −0.036]). All three hypothesized indirect pathways remained statistically significant after controlling for gender, demonstrating the robustness of the proposed sequential mediation model.

## 4. Discussion

College students mark a critical developmental stage during which individuals move from adolescence into adulthood, with certain limitations in their social practices and coping strategies. When faced with multiple pressures, they are highly susceptible to depressive mood characterized by low mood, lack of motivation, and confusion about life (S. M. Zhang et al., 2024). Without intervention, students may develop depression after experiencing a vicious cycle of stress and exhaustion. Extant literature has documented numerous individual-level risk factors for depression (M. Y. Wang et al., 2020). However, there remains a notable gap in research focusing on group factors, including school identification. Moreover, researchers have paid more attention to adolescents’ school identification (Verhoeven et al., 2019), and relatively few have directly investigated college students’ school identification. Given that social exclusion is a common interpersonal stressor among college students, and sense of control is a key resource for managing stress and mood, this study further explored how these two factors sequentially mediate the relationship between school identification and depressive mood to fill research gaps. Correlation analyses demonstrated that school identification was negatively related to depressive mood in college students. Thus, Hypothesis 1 was confirmed by the data. However, when social exclusion and sense of control were included as mediators, the direct effect of school identification on depressive mood was weak (Cohen, 1988). Its statistical significance should be interpreted with caution, as it may be partially attributable to the relatively large sample size. Furthermore, mediation analyses further identified social exclusion as a significant mediator between school identification and depressive mood, lending support to Hypothesis 2. Additional mediation analyses revealed that sense of control also served as a significant independent mediator, and the sequential mediating pathway through social exclusion followed by sense of control was also significant, thereby supporting Hypotheses 3 and 4. That is, school identification not only alleviates depressive mood through reducing social exclusion or enhancing sense of control separately, but also operates through a sequential pathway in which reduced social exclusion facilitates a stronger sense of control, which in turn alleviates depressive mood. Together, these mediating pathways form a serial mediation mechanism and jointly transmit the influence of school identification on depressive mood. The findings indicated that strengthening school identification can protect students from the harmful effects of social exclusion on sense of control, ultimately lowering their risk of depressive mood.

Second, social exclusion has been consistently linked to increased depressive mood, making it a critical contributing factor (X. L. Yang & Li, 2020). College students with higher school identification may reduce their sense of being excluded, thereby reducing their negative emotional experiences. Although considerable research supports the correlation between social identification and social exclusion (Shao et al., 2020), to the best of our knowledge, limited research has examined the mediating role of social exclusion in the relationship between school identification and depressive mood, and the present study addressed this gap in the existing literature. This study indicated that school identification significantly alleviated the negative impact (e.g., depressive mood) among Chinese college students through reducing social exclusion, at least in part. According to self-determination theory, individuals possess fundamental psychological needs for relatedness; when this need is unsatisfied, mental health is likely to be impaired (Deci & Ryan, 2013). School identification helps students integrate into groups and reduces the probability of social exclusion. Students with a high level of school identification recognize the school’s cultural values, develop a robust sense of group belonging, and establish good relationships with teachers and classmates, findings that align with prior research (Wei et al., 2024). These processes reduce the likelihood of social exclusion through three sequential mechanisms. First, at the cognitive level, high-identifying students internalize group norms and values, which reduces the attitudinal and behavioral distance from peers that often invites exclusion. Second, at the relational level, internalized identification facilitates the formation of supportive connections with teachers and classmates, thereby reducing the likelihood of being ostracized in academic and social settings. Third, at the behavioral level, these cognitive and relational foundations translate into active participation in campus activities and proactive academic planning, then concrete actions that enhance social embeddedness and further buffer against exclusionary experiences. These mechanisms are particularly salient for early-year college students, who face the transition from high school to university with their pre-existing support networks partially disrupted and new ones yet to be established. For these students, school identification serves as a psychological anchor that orients their initial social and academic behaviors, shaping their early trajectories of integration or withdrawal. Conversely, those who enter with lower identification—such as students who perceive themselves as having “deserved” a more prestigious institution—may be predisposed to psychological withdrawal, which in turn reduces their engagement and heightens vulnerability to depressive mood. Moreover, longitudinal evidence among Chinese early-year college students confirms that class belongingness exerts a stable negative predictive effect on depressive mood over time (H. L. Li et al., 2023), underscoring the protective function of school identification during this critical transitional period. Therefore, individuals who identify strongly with their school are less vulnerable to exclusionary experiences. By unpacking this pathway, the present study extends existing scholarship on school identification’s role in exclusion by identifying social exclusion as a key mechanism through which school identification shapes depressive mood in college students.

Sense of control is the belief that one can influence environmental outcomes. It is a robust psychological resource that protects individuals against depressive mood. Meta-analytic evidence has demonstrated a moderate-to-strong association between sense of control and depression, with a pooled effect size of *r* = 0.41 (Msetfi et al., 2024). Our findings demonstrate that sense of control independently mediates the relationship between school identification and depressive mood. This suggests that beyond reducing social exclusion, school identification also directly bolsters students’ sense of control to alleviate depressive mood. Social identity theory (Tajfel & Turner, 1979) provides a theoretical lens. Group identification offers cognitive resources, such as clear social categorization and role expectations. These resources render the environment more predictable and thus enhance sense of control. By providing a clear framework for understanding one’s place in the social world, school identification fosters a sense of mastery over academic and social life. In everyday practice, highly identified students are more likely to plan their coursework proactively, engage with campus resources, and set meaningful goals. They view their institution as a legitimate setting where personal efforts can yield positive outcomes. This behavioral pattern is a manifestation of enhanced control. By contrast, students who lack such identification may drift through university life without clear direction, completing assignments and attending classes passively. This passivity undermines their sense of control and heightens vulnerability to depressive mood.

A key finding of the current study was the significant sequential mediating effect of social exclusion and sense of control on the relationship between school identification and depressive mood, supporting Hypothesis 4. Two theoretical perspectives, when taken together, explain why reduced social exclusion facilitates enhanced sense of control. From the perspective of protection, the agency–communion model of social identity’s effects on depression (Yan et al., 2023) suggests that communion resources and agency resources both serve as protective factors against depressive mood. Within this framework, communion resources refer to social connectedness and a sense of belonging—being accepted, supported, and embedded in social relationships. Agency resources refer to the capacity to exert influence over one’s environment—the belief that one can shape outcomes and exercise personal control. The model suggests that both types of resources serve as protective factors against depressive mood, and they can operate independently or synergistically. From the perspective of risk, the temporal need-threat model of ostracism (Williams, 2009) explains why the absence of social exclusion is critical for maintaining control. This model posits that social exclusion does not only threaten belongingness, but also directly undermines individuals’ sense of control. Being ignored or excluded is inherently unilateral, leaving individuals unable to influence or escape the situation, which constitutes a direct assault on their perceived agency. Empirical evidence supports this link: experimental studies have demonstrated that social exclusion directly reduces individuals’ sense of agency and control (Malik & Obhi, 2019). Among Chinese college students, survey studies have further confirmed that social exclusion negatively predicts sense of control (Dai et al., 2025). In this sense, reducing social exclusion removes a direct threat to control, allowing it to be restored.

Integrating the above theoretical perspectives, the sequential pathway operates as follows: school identification reduces social exclusion, which in turn facilitates the development of sense of control, ultimately alleviating depressive mood. The reduction in social exclusion not only helps individuals gain communion resources but also establishes an interpersonal foundation that enables the development of agency resources—sense of control. In everyday campus life, students who experience less social exclusion have more opportunities to participate in collective activities and establish stable social connections, which provides a foundation for developing a sense of control over their campus environment. Those with a stronger sense of control are more likely to adopt proactive coping strategies, such as regulating their emotions and seeking external support, which helps alleviate depressive mood. The overall model accounted for 28.3% of the variance in depressive mood. The chain mediation pathway represented a non-negligible mediating mechanism, accounting for 18.33% of the total effect. The effect size of the sequential pathway (*b* = −0.049) was comparable to that reported in prior research on school identification and depressive mood among Chinese students (Yu et al., 2025). Thus, the pathway of “school identification → social exclusion → sense of control → depressive mood” captures a theoretically meaningful progression from social connectedness to personal mastery.

## 5. Limitations and Implications

While these results provide important implications for understanding depressive mood in relation to school identification among Chinese students, there are notable limitations to consider.

Firstly, the representativeness of the sample is limited. This study employed convenience sampling from a single university in China, with first and second-year students accounting for 93.2% of the total sample, whereas upper-year students were severely underrepresented. This imbalance limits our ability to fully examine whether the observed relationships hold consistently across different grade levels. Future research should employ more balanced grade samples and more diverse populations to further validate the stability of the model. Moreover, all participants were recruited from China, and the role of school identification may be shaped by the collectivistic cultural context. In Chinese culture, group belonging is closely intertwined with personal worth, and the protective effect of school identification on mental health may be more pronounced than in individualistic cultural contexts. Future research should examine the generalizability of our findings across a wider range of educational stages and more diverse cultural settings. Secondly, there are methodological limitations. This study adopted a cross-sectional design, which precludes causal inferences. All data were collected via self-report measures, raising concerns about social desirability bias and common method variance. Future studies could employ longitudinal or experimental designs, incorporate objective measures such as peer nominations, behavioral observations, or electrophysiological indicators, and standardize data collection methods to enhance the robustness of conclusions. Thirdly, the theoretical model leaves room for further expansion. This study only examined two mediators—social exclusion and sense of control—and other potential variables (e.g., meaning in life, personality traits, family environment) were not included. Future research could explore additional mediators or moderators to gain a more comprehensive understanding of the mechanisms through which school identification influences depressive mood.

Despite these limitations, this study makes several theoretical contributions. First, by integrating social cure theory, self-determination theory, and the agency-communion model, this study identifies three indirect pathways through which school identification alleviates depressive mood: via reduced social exclusion alone, via enhanced sense of control alone, and via the sequential pathway from social exclusion to sense of control. Critically, the serial pathway reveals that the protective effect of school identification operates through a temporal progression—first providing communal resources (reducing social exclusion), which in turn facilitates agentic resources (enhancing sense of control), and ultimately alleviating depressive mood. This finding provides empirical support for the agency-communion model’s core premise regarding the sequential interplay between communion and agency resources and extends its applicability from the domain of general social identity to the specific context of school identification among college students. Second, while previous research on depressive mood among college students has predominantly centered on individual-level psychological factors such as personality traits, cognitive styles, and attributional patterns, the present study adopts a group-level perspective by examining school identification as a protective resource. By documenting the mechanisms through which school identification operates, this study offers a clearer theoretical account of why group identification benefits mental health, showing that its effect unfolds through a cascading process that begins with social connectedness and culminates in enhanced personal control.

In addition, the present study yields clear practical insights into preventing depressive mood among university students. First, the results indicated that school identification is an important protective factor. A higher level of school identification means that college students have more abundant psychological resources, thereby reducing their depressive mood experiences. Existing evidence-based intervention programs primarily target first-year college students, enhancing their school belongingness through experiential group activities or social-belonging interventions (Costello et al., 2022; Walton & Cohen, 2011). Although these programs are designed for new students, their core logic, enhancing school-related emotional connectedness through fostering social connections, can inform school identification interventions for the broader college student population. Schools may consider incorporating student interests into curriculum teaching (Virtanen et al., 2021), organizing orientation activities such as campus tours and peer mentoring, and fostering a supportive campus cultural environment to strengthen students’ sense of identification. Although mature evidence-based interventions specifically targeting school identification remain limited, some researchers have begun to conduct exploratory studies on school identification interventions (Zhu, 2025). Future research could build on these foundations and further examine the effectiveness of school identification interventions through randomized controlled trials. School administrators may draw on the design principles of existing intervention programs and adapt them to their institutional contexts to systematically enhance students’ level of school identification.

Second, social exclusion and sense of control serve as two key pathways linking school identification to depressive mood, and they also form a sequential pathway. These findings offer multi-level practical implications for prevention and intervention. At the school level, reducing social exclusion should be a priority. Schools can strengthen teacher-student and peer interactions to enhance students’ sense of belonging. For example, S. M. Cheng et al. (2022) organized activities in which students shared their experiences of overcoming loneliness, which helped break cognitive biases and enhanced their sense of belonging and self-confidence. Such practices may reduce perceived social exclusion and, in turn, alleviate depressive mood. Beyond reducing exclusion, schools should also attend to the cultivation of students’ sense of control. Schools may provide students with more opportunities to make their own choices, help them set achievable goals, and allow them to experience the outcomes of their own decisions. At the family level, parents’ continued involvement in and care for their children’s lives, as well as fostering their interpersonal and emotion-regulation skills, can also contribute to consolidating children’s sense of control. Moreover, a staged approach in school practice is warranted. Schools should first create an inclusive campus environment to reduce students’ experiences of social exclusion, and then build on this foundation to further cultivate their sense of personal mastery. If students continue to suffer from exclusion, interventions that focus solely on enhancing sense of control may have limited effectiveness in alleviating depressive mood.

In summary, the present study identifies three indirect pathways—via reduced social exclusion alone, via enhanced sense of control alone, and via their sequential combination—through which school identification exerts its protective effect against depressive mood among college students. The findings demonstrate that school identification does not merely supply psychological resources in an additive manner. Social exclusion and sense of control can function independently, and they can also form a progressive relationship, whereby individuals develop a sense of personal mastery based on social connectedness. Schools can foster an inclusive campus environment to reduce students’ perceptions of social exclusion and attach importance to cultivating students’ sense of control, so as to safeguard college students’ mental health through multiple approaches.

## Figures and Tables

**Table 1:** Descriptive statistics and interrelations among all of the observed variables.

Variables	*M*	*SD*	1	2	3	4	5	6
1. Gender	1.530	0.500	1					
2. Grade	1.360	0.763	0.131 **	1				
3. School identification	4.507	0.962	0.059	−0.029	1			
4. Social exclusion	2.617	0.561	−0.094 **	−0.001	−0.389 **	1		
5. Sense of control	4.426	0.771	0.130 **	−0.001	0.300 **	−0.455 **	1	
6. Depressive mood	1.576	0.582	−0.114 **	0.025	−0.275 **	0.425 **	−0.468 **	1

*Note*. *N* = 981. ** *p* < 0.01.

**Table 2:** Effect sizes and Bootstrap analysis results of mediating effect.

Path	Effect	Percentage	95% CI	
			LL	UL
School identification → Social exclusion → Depressive mood	−0.082	34.14%	−0.125	−0.063
School identification → Sense of control → Depressive mood	−0.047	17.51%	−0.074	−0.023
School identification → Social exclusion → Sense of control → Depressive mood	−0.049	18.33%	−0.065	−0.036
Total effect	−0.270	100%	−0.330	−0.210
Total indirect effect	−0.189	70%	−0.231	−0.150

## Data Availability

Due to ethical restrictions and participant privacy concerns, thedatasets generated and analyzed in this study are available from the corresponding author upon reasonable request.
